# DNA Organization along Pachytene Chromosome Axes and Its Relationship with Crossover Frequencies

**DOI:** 10.3390/ijms22052414

**Published:** 2021-02-27

**Authors:** Lucía del Priore, María Inés Pigozzi

**Affiliations:** INBIOMED-Instituto de Investigaciones Biomédicas, Universidad de Buenos Aires-CONICET, Facultad de Medicina, Paraguay 2155, C1121ABG Buenos Aires, Argentina; luciadelpriore24@gmail.com

**Keywords:** meiosis, crossing over, synaptonemal complex, MLH1 focus map, bird chromosomes, recombination frequencies, immunostaining, fluorescent in situ hybridization, molecular cytogenetics

## Abstract

During meiosis, the number of crossovers vary in correlation to the length of prophase chromosome axes at the synaptonemal complex stage. It has been proposed that the regular spacing of the DNA loops, along with the close relationship of the recombination complexes and the meiotic axes are at the basis of this covariation. Here, we use a cytogenomic approach to investigate the relationship between the synaptonemal complex length and the DNA content in chicken oocytes during the pachytene stage of the first meiotic prophase. The synaptonemal complex to DNA ratios of specific chromosomes and chromosome segments were compared against the recombination rates obtained by MLH1 focus mapping. The present results show variations in the DNA packing ratios of macro- and microbivalents and also between regions within the same bivalent. Chromosome or chromosome regions with higher crossover rates form comparatively longer synaptonemal complexes than expected based on their DNA content. These observations are compatible with the formation of higher number of shorter DNA loops along meiotic axes in regions with higher recombination levels.

## 1. Introduction

The synaptonemal complex (SC) is an evolutionarily conserved structure that has been found in most sexually reproducing organisms. The SC is formed by a “zipper”-like protein assembly that synapses homolog pairs together and provides the structural framework for processing recombination sites into crossovers reviewed by the authors of [1]. The SC consists of two lateral elements, a central region or central element and transverse filaments that extend between each lateral element and the central element [2]. Lateral elements of the SC develop from the axial elements or chromosomal axes that appear during leptotene and contain coiled-coil domain proteins, cohesin complexes, and the bases of large loops of genomic DNA [3,4]. In addition to serve as anchoring sites for loops of chromosomal DNA, meiotic axes provide support for the protein complexes that initiate recombination [3,5]. As a consequence of the axis association of recombination complexes, recombination initiation and frequency are tightly related to axis length [6,7]. Interspecies comparison of synaptonemal complex length, DNA content and crossing over shows that SC length/DNA ratios are positively correlated with crossover frequencies in higher plants and vertebrates; that is, organisms with higher CO rates have proportionally longer synaptonemal complexes [8,9]. Since the SC structure has limited variation, it was proposed that this relationship depends on the spacing of DNA/chromatin loops along the chromosome axes and that the loop density is a conserved feature across species. As a result, for a given genome size, chromosomes with longer loops have shorter axes while chromosomes with shorter loops have longer axes [6,10]. Considering the role of axis length in determining the frequency of CO events, longer/shorter loops imply shorter/longer axes, which in turn imply fewer/more COs [11]. A great deal of this argument is based on estimates of DNA loop length in micrographs of pachytene nuclei. The loop length in micrometers is converted to DNA base pairs to obtain the amount of DNA per loop. The haploid DNA content of an organism divided by the estimated size of a single loop will give the number of loops that could potentially form along the synaptonemal complexes. [12,13]. However, these measurements are not precise due to the “halo” appearance that adopts the chromatin in meiotic spreads and because longer loops can mask the shorter ones. Even if chromosome painting is used to highlight the loops in pachytene spreads, different conditions in image capture can introduce substantial error in the calculation of DNA bp per loop. Support for this caveat comes from the difference in loop sizes, up to one order of magnitude, obtained by cytological vs. genomic methods in mouse pachytene spermatocytes [12,14]. These methodological variations ultimately affect the conclusions over the relationship between number of loops, axis length, and CO frequencies.

In an attempt to circumvent this problem, we present here a different approach to investigate how the DNA organization along meiotic axes and CO frequencies are related. It consists of calculating the SC/DNA ratio at specific chromosome segments and contrast this ratio with cytological data of crossing over in the same segments. As genome sequencing projects advance, complete or nearly complete assemblies of individual chromosomes become available to obtain the distance in Mb between specific single-copy sequences. The same sequences can be localized using fluorescent in situ hybridization (FISH) on pachytene chromosomes to measure the physical distances along the SCs. With both parameters, it should be possible to determine the DNA density per unit of SC length at specific segments of a bivalent and, conversely, the SC/DNA ratio. We employed chicken SC spreads since they are well-established as experimental systems for quantitative analysis of meiosis and they are good substrates for in situ hybridization in combination with immunostaining [15,16,17,18]. In the last version of the chicken genome assembly, most linkage groups—especially macrochromosomes—are assembled at chromosome level with wide coverage and few genomic issues [19]. The crossover data were obtained by immunolocalization of the protein MLH1 in chicken oocytes at pachytene. MLH1 protein is a cytological marker of crossing over that localizes in “foci” on SCs of birds and other vertebrates [20,21,22,23]. The MLH1-focus map can be directly overlapped on the same SC segments investigated for SC/DNA ratios to observe how the DNA organization along the SCs and the recombination frequencies relate to each other.

Our present results demonstrate different DNA packing ratios between the largest bivalents and microbivalents in line with the differences in crossover rates. It is also shown that the microbivalents and a terminal segment of the largest bivalent have longer synaptonemal complexes than expected according to the amount of attached DNA. At the same time these chromosomes/chromosome segments have higher CO rates, showing the coordinate variation of the DNA organization and crossing over.

## 2. Results

### 2.1. DNA Densities in Macro- and MicroSCs

**Figure 1:**
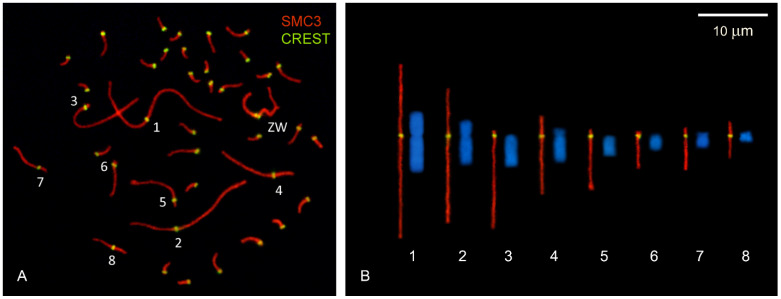
Mitotic chromosomes and synaptonemal complexes (SCs) in the chicken. (**A**) Surface spread of a pachytene oocyte. The SCs are labeled in red using an antibody against the cohesin SMC3. The centromeres were localized using human CREST serum and detected in green. The numbers next to the centromere signals mark the eight largest bivalents, which can be identify by their relative lengths and the centromere position. (**B**) Comparison of the mitotic macrochromosomes of the chicken with their respective SCs. The first eight autosomal SCs of the oocyte in (**A**) were digitally straightened to show the morphological correlation with the mitotic chromosomes. Bar: 10 μm.

**Figure 2:**
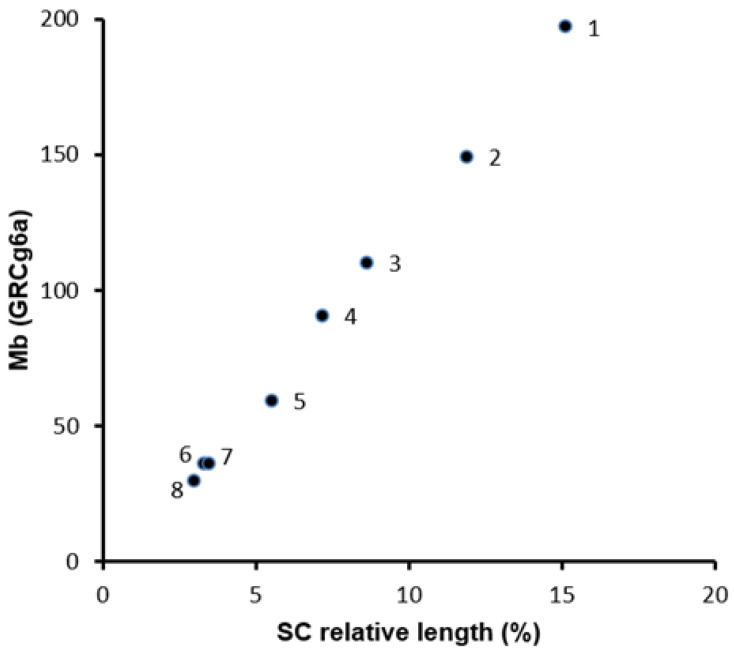
Relationship between macrochromosome sizes and SC length. The size of each macrochromosome in the genome assembly (galGal6) are in Mb; the SC lengths are expressed as a percentage of the total SC set. A regression test show that both parameters are significantly correlated (Pearson regression test, r = 0.988; *p* < 0.0001).

The absolute and relative lengths of complete SC sets and the eight largest SCs (macroSCs) were obtained from 138 immunostained oocyte nuclei at pachytene (Figure 1A). In each spread nucleus, the macroSCs can be identified by size and centromeric index and, based on this data, assigned to a specific somatic chromosome pair (Figure 1B). Measurements from individual nuclei also show that the SC corresponding to GGA1 (SC1) is always the largest and, therefore, it can be identified solely by length in pachytene spreads. There is a positive linear relationship between the chromosome sizes in Mb and the macroSC relative lengths (Figure 2; Table 1). Taking into account the concordant centromeric indexes of SCs and mitotic chromosomes, we estimated the position of the centromere of GGA1 at the Mb scale using the percent represented by the short arm on SC1. The average relative position of the centromere signal on SC1 is 0.41 of the total SC length placing the centromere at 81 Mb from the sequence start. In the GRCg6a genome model, the centromere of GGA1 was arbitrarily assigned as a stretch of 500,000 Ns located between 76.6 to 77.1 Mb from the sequence start (https://www.ncbi.nlm.nih.gov/genome/gdv/?org=gallus-gallus). Data from karyological markers combined with the location of the main CENP-A peaks in chromosomes place the centromere of GGA1 around 74.6 Mb from pter [24]. The difference with our present results might be related to local variations of the DNA organization along SC1, especially at the terminal region of the short arm (see next section).

We found that the average linear amount of DNA or DNA density per µm of SC in *G. domesticus* is 6.1 Mb/µm with densities above the average in the largest SCs (Table 1). Although specific microbivalents were not identified in spreads, the DNA density calculated for SCs 9–38 is below the average for the whole SC set. Microbivalents show at least one MLH1 focus, in line with the need to form one obligatory CO to ensure proper segregation. The averaged CO rate for the microSC set, based of MLH1 focus counts, is up to three times higher compared to that of macroSCs (Table S3; Figure S7). At the same time, DNA density is lower in microSCs, and consequently they have higher SC/DNA ratios. As discussed below, these findings support the interdependence between SC length, DNA content and crossing over.

### 2.2. DNA Density at Specific Intervals of SC1

**Figure 3:**
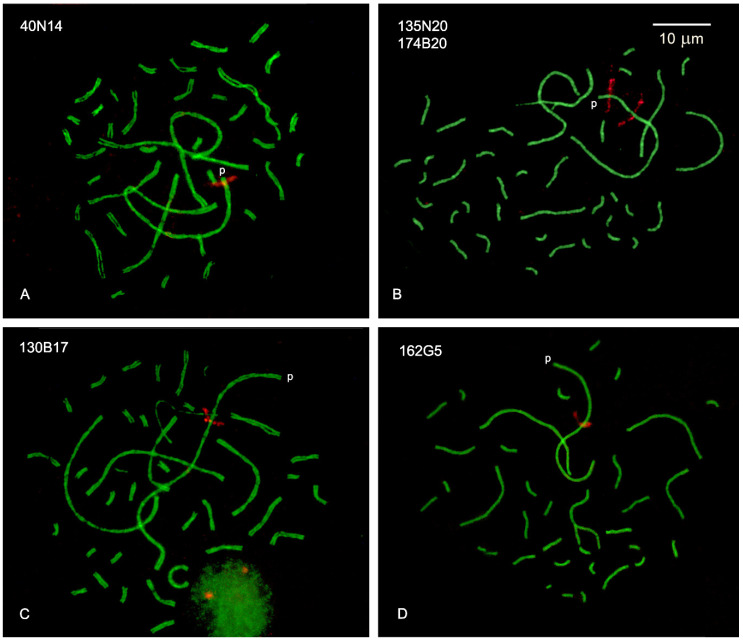
Localization of BAC clones on SC1 using FISH. (**A**–**D**): the SCs are labeled in green and the signal of the fluorescent in situ hybridization (FISH) probes in red. Each image shows the location of one or two BACs on SC1. The BAC IDs are shown in each image at the top left corner. The end of the short arm (p) can be identified by the position of the FISH signals which are asymmetric respect to the SC termini. Bar: 10 μm.

**Figure 4:**
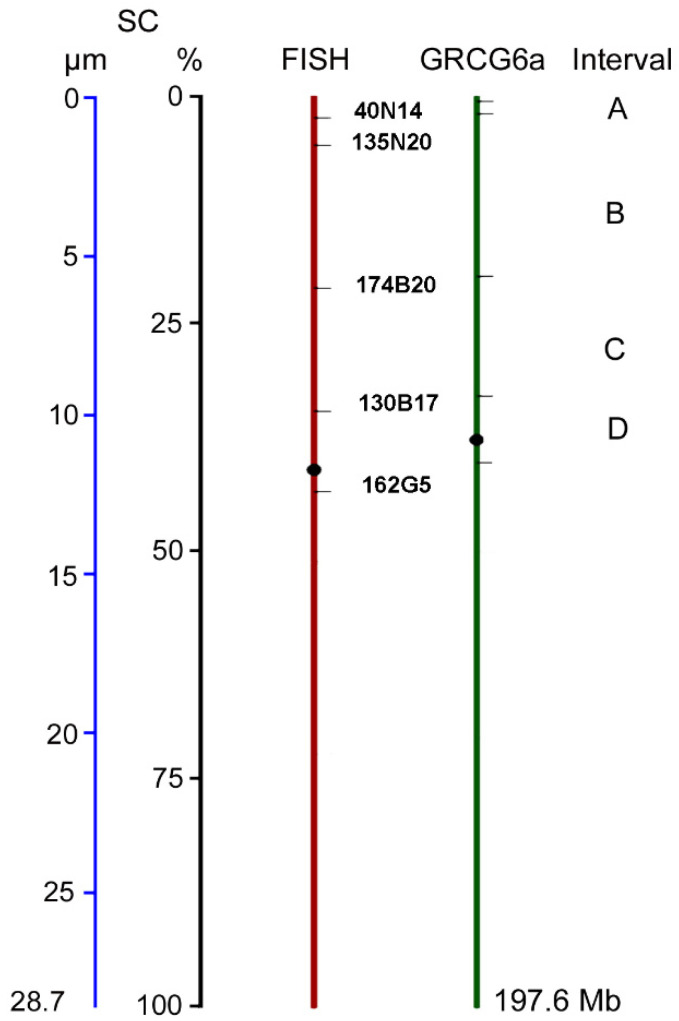
Comparative positions of the BAC clones on the SC and the GGA1 assembly (GRCG6a). The average position of the FISH signals on SC1 (red line) are compared to the relative positions in the chromosome assembly (green line). For the signals on the SC both, absolute (μm) and relative (%), positions are shown, with the scales for these positions depicted in blue and black, respectively. The positions of the BAC-clones are shown as relative positions respect to the whole length of GGA1 assembly (197 Mb). The letters A–D are the intervals between BACs. Numerical data for this representation can be found in the Supplemental online material (Table S2).

We used the location of the BAC clones on GGA1 and their relative positions along the SC to delimit four intervals and then calculate the DNA density (bp/μm) at these specific chromosomal segments. Examples of pachytene spreads after Bacterial Artificial Chromosome clones localized by Fluorescent in situ Hybridization (BAC-FISH) and immunostaining of SCs are in Figure 3. The location of the target sequences along SC1 was defined first in single FISH experiments; double-FISH was used in subsequent experiments to increase the number of nuclei for measurements. The selected BAC clones had asymmetric positions relative to the chromosome ends in the genome assembly (Figure S1) and the BAC FISH signals were concordant with this asymmetry helping to pinpoint the end of the short arm for chromosome orientation (Figure 3). The location of each BAC signal was scored on 7 to 14 SC spreads and then expressed as a percentage of the SC length, to obtain an average position on the linear SC (Table 2; Figure S1). BAC-FISH measurements were very consistent with SDs of 0.5 µm or less for the final position of each BAC and they are positively correlated with the relative positions on GGA1 assembly (Table 2; Figure S3). A comparative diagram of the positions calculated from BAC-FISH and those relative to the chromosome size in the GRCg6a build is presented in Figure 4. The relative positions of the FISH signals appear more distant from pter than those calculated from the GGA1 assembly. Part of this variation may be ascribed to the fact that terminal sequences such as telomeric repeats and GC-rich subtelomeric sequences are not included in the assembly and therefore do not contribute to the distances calculated from genomic data. For this reason, we did not use the terminal SC segment, distal to the first probe, in the calculations of DNA densities along the SC.

The SC segment between the first and the last BAC-FISH signals comprises most of the short arm and a small pericentromeric region on the long arm. Its average size, calculated from the average positions of the first and last BAC-FISH signals is 11.6 µm (Table 2). According to the GGA1 assembly, this region contains 78.4 Mb of linear DNA. From these values, the region between the localized BACs has an average DNA density of 6.8 Mb/µm which is very similar to the average for the whole SC (Table 1). From the average positions of the five BAC signals and the Mb size of each interval between adjacent BAC clones, it is possible to determine de DNA density in four intervals (Figure 4). In three of these intervals the DNA densities differ by 16 percent or less from the average, but at interval A, the DNA density is more than 50% lower (Table S2). To determine if the mean DNA densities differ at statistical level between intervals, we calculated the DNA densities in all the possible intervals using the signals of adjacent probes as the lower and upper limit of each interval (Figure S2A,B). The mean DNA densities show statistically significant differences as determined by one-way ANOVA [F (3, 570) = 1339; *p* < 0.0001]. The means are also statistically different if data for interval A are excluded from the analysis [(F (2, 473) = 445,8; *p* < 0.0001)]. It can be inferred that the DNA densities vary in different segments of the SC, being in some cases higher and in other cases lower than the average expected for the entire bivalent. These variations ultimately compensate each other, explaining the similarity between cytological and genomic positions of the BACs.

### 2.3. Crossover Rates and SC/DNA Ratio at Different Intervals on SC1

**Figure 5:**
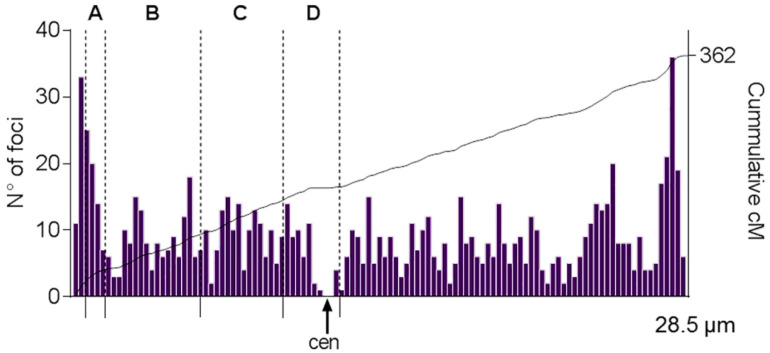
Crossover distribution along SC1. The histogram represents the distribution of MLH1 foci along the largest synaptonemal complex in chicken oocytes. The *x*-axis indicates the positions of the MLH1 foci on the SC. The end of the short arm is at 0 and the end of the long arm at 28.5 which is the average length of the SC1. The bin width is equivalent to 0.25 μm. The height of each bar indicates the number of foci observed at a given interval. The continuous line is the cumulative cM along the SC1 calculated from the number of MLH1 foci in 138 pachytene nuclei. SC1 has on average 7.2 MLH1 foci, so the total genetic length of this bivalent is 362 cM (number of crossovers multiplied by 50). The dashed lines mark the average positions of the five BAC-FISH signals. The four intervals delimited by the BAC clones are labeled A to D. cen: centromere position.

MLH1-focus positions along SC1 were recorded from a sample of 138 immunostained pachytene nuclei, to obtain a frequency histogram where the *X*-axis represents the average SC length divided into 0.25-μm intervals and the height of each bar shows the number of MLH1 foci observed per interval (Figures S4 and S5; Figure 5). A total of 1000 MLH1 foci were scored in this sample, which gives an average of 7.2 foci. Since each MLH1 focus represents a genetic distance of 50 cM, the genetic distance in cM in a given interval is obtained by multiplying the average frequency of foci in a chromosomal segment by 50. The total genetic length for GGA1 based on MLH1-focus counts is then 362 cM (7.2 × 50). The genetic size of each the four intervals (A to D) defined by the BAC-FISH was obtained from the number of foci within each interval (Table 3). As expected, the genetic length varies -from 21 to 57 cM- and these variations are independent of the linear amount of DNA in the interval. For example, intervals C and D differ by 10 Mb in size but they have similar genetic lengths. The CO rates instead are 5–6 times higher at interval A compared to the other intervals. As previously explained, this interval also has the highest SC/DNA ratio (Table 3), adding support to the idea of interdependence between DNA organization along meiotic axes and CO frequencies.

## 3. Discussion and Conclusion

The present results show the existence of differences in the DNA packing ratio in chicken pachytene chromosomes, both at intra- and interchromosomal levels. This finding is of importance to understand the relationship between the DNA organization and the regulation of crossover frequencies during the first meiotic prophase. It is known, from the comparison of mitotic vs. meiotic (pachytene) chromosome lengths that the SC is under-represented in highly compact chromatin, that is, the DNA density per µm of SC is higher in heterochromatin versus euchromatin. This evidence comes from a variety of organisms such as tomatoes, mice and humans and lead to the hypothesis that DNA loops along SCs are regularly spaced, with the consequence that the degree of chromatin compaction is reflected in the length of the SC, as smaller loops would yield longer SCs [25,26,27,28,29,30,31]. Conversely, the same amount of linear DNA organized in longer loops would result in shorter chromosomal axes [11,32]. In the chicken and most avian karyotypes, heterochromatin is mainly confined to microchromosomes [33], thus the lower DNA density found here in microbivalents seems to contradict the general view over the DNA arrangement in heterochromatin vs. euchromatin along meiotic axes. However, a thorough look at cytogenetic data shows that only the five shortest chicken microchromosomes are fully heterochromatic, while the rest of midi- and microchromosomes only have heterochromatin at centromeres [34,35]. Therefore, most DNA in chicken microchromosomes is confined to euchromatin, explaining their lower DNA density compared to macroSCs. Variations in the amount of linear DNA per unit of SC length was inferred from the differential effects in G bands relative to R bands in mammalian chromosomes reflected in discrepancies between pachytene and mitotic metaphase chromosomes [23,36,37]. However, local variations of DNA packing at specific SC segments of individual pachytene bivalents were explored only in tomato heterochromatin and at mouse telomeric regions [30,38]. The present results show variations of the amount of DNA per SC unit length (Mb/μm) between the distal and interstitial regions of the short arm of the SC1. At a subtelomeric segment, the amount of DNA is lower than the average calculated for the whole SC arm. It means that, at this subtelomeric segment, the SC is longer than expected based on its DNA content. It has been proposed that in the absence of modifications of the axial structure, differences in total genome size among different organisms are accommodated by differences in loop size or total axis length [28,39]. Electron microscopy and immunostaining analyses, and a sequence study of SC proteins in the chicken demonstrate that the structure and composition of SCs in birds are highly conserved with mammals [40,41]. In the chicken, the lateral elements show a uniform appearance with both, electron and structured illumination microscopy [42,43], suggesting that the protein components are not differentially organized throughout the SC length. For the same amount of DNA, a longer SC segment is needed to attach shorter than longer loops. The base composition of the DNA also influences meiotic axis length, as the GC content in a particular chromosome is positively correlated with the relative SC length. For example, in human spermatocytes, chromosomes with higher GC content and gene density have SCs with a relative length longer than the mitotic relative length [44]. According to the default tracks in the UCSC Genome Browser on chicken assembly GRCg6a, the interval A (as defined here using BAC-FISH) on GGA1 has a GC percent of 46 compared to 39–41 in the other three intervals (Figure S6). A relationship between GC content and SC length can also be observed for micro-SCs. Chicken microchromosomes are GC rich and gene dense [45,46,47], and here it was shown that they form longer SCs than expected based on their DNA content. In mice, the loops attached to the SC in the telomeric and subtelomeric regions are smaller [38], and they are known to be GC- and gene-rich [48]. Further, a recent association study of recombination rates and isochore composition at topologically associated domains (TADs) suggests that the DNA loops along mouse meiotic axes are shorter at GC-rich DNA regions because they have higher bendability and are therefore capable to form shorter loops compared to AT-rich regions [49]. In the absence of longitudinal variations of the SC structure, the higher SC/DNA observed at the distal, subtelomeric segment of the chicken SC1 should respond to the formation shorter DNA loops compared to the other intervals on the same bivalents. The GC-rich nature of the DNA sequence of micro-SCs and the terminal SC1 interval can provide sufficient flexibility to allow the formation of shorter loops.

As previously explained, the higher SC/DNA ratio in microbivalents and the subtelomeric interval of SC1 could be achieved by the formation of shorter DNA loops at these chromosomes or chromosome region. Because CO rates are higher in microSCs and the mentioned interval of SC1, we interpret that our findings in chicken oocytes provide further evidence of a relationship between DNA organization along SCs and the recombination rates. It has been proposed that the link between these two variables is the tight association of the recombination complexes to chromosome axes with the consequence that the distance metric for recombination is the physical distance along a prophase chromosome [11,38]. Consequently, longer meiotic axes would anchor a large number of shorter DNA loops, thus increasing the chances of forming more DSBs that start the CO interactions [10,13,50]. Experimental evidence supporting this hypothesis relies on the estimation of loop length from electron microscopy images or using FISH on synaptonemal SC spreads to calculate the number of DNA loops attached per unit of SC length. It should be pointed out, however, that in mouse spermatocytes the loop size estimated by these morphological methods is about 180 kb [12], considerably lower than the 2 Mb found in the same species using chromosome conformation capture [14]. Due to these variations, it is difficult to establish a precise relationship between the size of the DNA loops along the SC and the CO rates. Here, the relationship between DNA organization and CO rates was established comparing the SC/DNA ratio at chromosome regions with known recombination frequencies in chicken oocytes. It was established that chromosomes or chromosome regions with higher CO rates show higher SC/DNA ratios or, in other words, the SC is longer than expected based on the amount of attached DNA. The present results are compatible with the hypothesis of the regular spacing of DNA loops along the meiotic chromosome axes. Considering the conserved nature of the DNA loop organization along the SCs, the present conclusions can be extended to other organisms.

## 4. Materials and Methods

### 4.1. Biological Material and Synaptonemal Complex Spreads

SC spreads from oocytes of *Gallus domesticus* (2n = 78) were obtained from white commercial layer females around hatching day. Handling and euthanasia of birds were performed according to protocols approved by the Animal Care and Use Committee of the University of Buenos Aires School of Medicine (EXP-UBA 0049207/16, Res 2061/16) following all institutional and national guidelines for the care and use of farm and laboratory animals.

### 4.2. Immunostaining and BAC-FISH

The methods for spreading meiotic cells as well as the combination of immunostaining and FISH on avian SC spreads were described previously [18]. The following antibody combination was employed for immunodetection of SCs, centromere proteins, and MLH1: rabbit anti-SMC3 (at 1:1000, Merck KGaA, Darmstadt, Germany), mouse anti-MLH1 (at 1:30, BD Pharmingen, Franklin Lakes, NJ, USA) and CREST serum of human origin (at 1:100, Roquel Laboratories, Buenos Aires, Argentina). The secondary antibodies (Jackson ImmunoResearch, West Grove, PA, USA) were: FITC or TRITC-labeled goat anti-rabbit, Cy3- or FICT-labeled donkey anti-human and FITC-labeled goat anti-mouse. The BAC clones were obtained from the BACPAC Resources Center (https://bacpacresources.org/, Emeryville, CA, USA). These BACs have unique and concordant placements on chromosome 1 (GGA1) in the current red jungle fowl (*Gallus gallus*) assembly, GRConsortium Chicken Build 6a. Further details on BAC IDs, insert size and their positions from the sequence start can be found in the Supplementary Material. To prepare the probes for FISH, the BAC DNA was extracted with a miniprep kit (Zymo Research, Irvine, CA, USA), quantified using a NanoDrop spectrophotometer, and labeled by nick-translation with biotin-16-dUTP (Roche Applied Science, Mannheim, Germany) according to the manufacturer instructions. About 50 ng of probe were used for each slide followed by overnight incubation at 37 °C. Stringent washes and the simultaneous detection of SMC3 and the probes were done as described previously [18]. Preparations were scanned with a PlanNeofluar 100× magnification objective at a fluorescence microscope (Zeiss Axioscope, Carl Zeiss, Jena, Germany) equipped with appropriate filter sets for each fluorochrome and HBO lamp illumination system. Individual images for red and green fluorescence were acquired using an Olympus DP73 CCD camera, corrected for brightness and contrast, and merged using Adobe Photoshop CS5 (Adobe Systems Inc, San Jose, CA, USA). The location of each FISH signal on SC1 was registered respect to the telomere of the short SC arm (pter) and then expressed as a fraction dividing the distance by the total SC length in each particular nucleus [18]. To express the BAC locations in micrometers, the relative position of each BAC signal was multiplied by the average absolute length of SC1. Datapoints can be found in Data file 2 at Mendeley Data, V1, doi:10.17632/w3n9xp5dnp.1.

### 4.3. MLH1-Focus Map along SC1

The construction of crossover frequency histograms from MLH1 foci in birds and other vertebrates is fully explained in several publications [51,52]. Briefly, individual SC lengths, centromeric signals, and MLH1 foci were scored using version 3.3 of the MicroMeasure program [53] which records absolute and relative distances on digitized images. To generate the recombination maps of the SC1, we calculated the absolute position of each MLH1 focus by multiplying the relative position of each focus by the average absolute length of the chromosome arm. These data were pooled for each arm and graphed in histogram form. The genetic map length in cM of each interval in this histogram (bin size 0.25 µm) was calculated multiplying the average number of MLH1 foci by 50. To produce the cumulative cM distribution, the average number of MLH1 foci per interval was converted to cM and then added along the SC starting from the tip of the short arm [54]. Individual SC measurements and MLH1 focus positions along SC1 are openly available at Mendeley Data, V1, doi:10.17632/w3n9xp5dnp.1, Data file 3.

## Figures and Tables

**Table 1:** SC lengths, chromosome sizes and DNA density in *Gallus domesticus*.

	Chr Size (Mb) ^a^	SC Spread Parameters		DNA Density	
Chr	GRCg6a	µm	RL (%)	CI	Mb/µm
**1**	197.7	28.7	15.3	0.41	6.9
**2**	149.7	22.2	11.8	0.35	6.7
**3**	110.8	16.2	8.6	0.04	6.8
**4**	91.3	13.5	7.1	0.24	6.8
**5**	59.8	10.3	5.4	0.09	5.7
**6**	36.4	6.1	3.3	0.08	6.0
**7**	36.7	6.5	3.5	0.24	5.6
**8**	30.2	5.6	3.0	0.38	5.4
**9–38**	436	78.5 ^b^	42	-	5.5
**Total**	1148 ^c^	187.6	-	-	6.1

^a^ The chromosome sizes in Mb are from the GRConsortium Chicken Build 6a. ^b^ MicroSC set = Total autosomal SC set (187.6 µm)–MacroSC set (109.1 µm). ^c^ Autosomal Mb content = total haploid DNA (1230 Mb)–Z chromosome (82 Mb); data from Warren et al., 2017.

**Table 2:** Positions of the BAC clones along GGA1 (GRCg6a) and the SC1 (BAC-FISH).

	GRCg6a		BAC-FISH ^c^			
BAC ID	bp ^a^	% ^b^	%	SD	µm	SD
**40N14**	1,274,288	0.6	2.5	0.3	0.7	0.1
**135N20**	4,069,830	2.0	5.5	0.5	1.6	0.1
**174B20**	39,215,363	19.8	21.1	0.3	6.0	0.2
**130B17**	64,984,575	32.8	34.4	1.0	9.8	0.3
**162G5**	79,454,082	40.2	43.4	0.8	12.3	0.3

^a^ Distance in bp from the sequence start to the mid-point of the BAC. ^b^ Relative position = Position in bp/Total chromosome size. ^c^ Average relative (%) and absolute (µm) distances from pter to the FISH signals.

**Table 3:** Relationship between SC/DNA ratio and crossover rates at specific intervals on GGA1.

Interval ^a^	SC Length (µm)	Size (Mb)	SC/DNA Ratio	cM	CO Rate (cM/Mb)
A	0.9	2.8	0.31	29	10.3
B	4.4	35.1	0.13	57	1.6
C	3.9	25.8	0.15	52	2.0
D	2.5	14.5	0.17	21	1.4

^a^ Each interval is the segment of SC (µm) or chromosome assembly (Mb) between adjacent BACs. Interval limits and sizes can be found in the Electronic Supplementary Material.

## Data Availability

The data presented in this study are openly available at Mendeley Data, V1, doi:10.17632/w3n9xp5dnp.1.

## Supplementary Materials

Supplementary Materials can be found attached to the present submission. The following are available online at https://www.mdpi.com/1422-0067/22/5/2414/s1, Figure S1. Positions of the BAC clones on the chromosome 1 (GGA1) assembly, Figure S2. DNA density at different intervals along SC1, Figure S3. Relative distances of the BAC clones on SC1, Figure S4. Spread chicken oocytes immunostained for SCs and MLH1, Figure S5. Additional typical pachytene nuclei used for MLH1 focus counts, Figure S6. GC content plot of GGA1 1-80, Figure S7. Chromosome size and CO rates are inversely related, Table S1. Positions of the BAC clones expressed in bp from the sequence start of the assembly of GGA1, Table S2. DNA density in the intervals limited by the BAC clone positions on the SC, Table S3. Average number of foci in macro- and microSCs.

## Abbreviations

SC	Synaptonemal complex
CO	Crossover
BAC-FISH	Bacterial Artificial Chromosome clones localized by Fluorescent in situ Hybridization
