# Supplementary material for: DNA Organization along Pachytene Chromosome Axes and Its Relationship with Crossover Frequencies
Source: Int J Mol Sci. 2021 Feb 27;22(5):2414. doi: 10.3390/ijms22052414 (PMC7957551; doi:10.3390/ijms22052414)
Supplement: Supplementary file 1 [file ijms-22-02414-s001.pdf]

Electronic Supplementary Material

BAC clones used for FISH and their positions on the GGA1 pseudomolecule

Five BAC clones were localized using FISH on synaptonemal complex spreads of chicken oocytes. Figure S1 shows that these BACs have unique and concordant placements on chromosome 1 (GGA1) in the current red jungle fowl (*Gallus gallus*) assembly, GRConsortium Chicken Build 6a (GRCg6a).

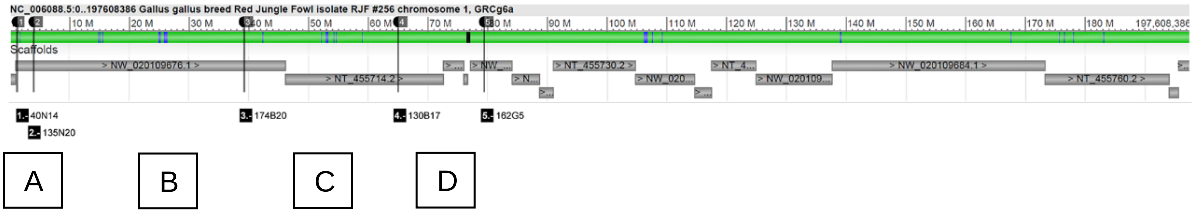

Figure S1. Positions of the BAC clones on the chromosome 1 (GGA1) assembly. BACs were located using the search function of Genome Data Viewer and the image of the assembly was downloaded from the browser. The vertical lines signal the position of each BAC. The BAC IDs were replaced by numbers on the top of each line to avoid overlapping with the Mb scale. Four of the BAC clones are located on the short arm and #5 is on the long arm. The position of the centromere is between 76-77 Mb from the sequence start and it is represented in black line on the sequence. The sequence representation is in green and the contigs are in grey.
The intervals between BAC clones/probe signals probes are named A, B, C and D throughout the paper.

For each BAC the mid-point was used to calculate their relative positions in the chromosome assembly (GGA1). The mid position is the start end plus the BAC size/2; the relative position of a given BAC is then the mid-position in Mb divided by the total chromosome size (197.6 Mb). The midpoint of each BAC was used to calculate its relative position on GGA1. Data for these calculations can be found in Table S1.

Table S1. Positions of the BAC clones expressed in bp from the sequence start of the assembly of GGA1

BAC ID	start	end	Insert size	Midpoint [a]
40N14	1,140,234	1,408,342	268108	1,274,288
135N20	3,964,778	4,174,881	210104	4,069,830
174B20	39,126,034	39,304,690	178657	39,215,363
130B17	64,873,862	65,095,288	221426	64,984,575
162G5	79,353,326	79,554,837	201511	79,454,082

[a] Amount of linear DNA in bp from the sequence start to the mid-point of each BAC

Comparison of SC/DNA ratios between intervals on SC1

Because all five probes were not used simultaneously, we generated all possible 574 intervals between adjacent probes using the individual absolute positions of probe signals as the interval´s lower and upper limits (deposited at Mendeley Data, V1, doi: 10.17632/w3n9xp5dnp.1. Data file 2: Absolute distance columns). To obtaien the average size of interval A, 98 distances (or interval sizes) were generated from each BAC-FISH position for 135N20 minus each position for 40N14, which are the limits of this interval (See Figure S1). The average size of each interval can be found in with an average size of 0.9 µm (Table S2; Table 3, main text). For each individual interval the DNA density was calculated dividing the size of each interval in Mb by its size in µm. Figure S2 and Table S2 summarizes the DNA density for the generated intervals and the comparison of the interval means using one-way ANOVA.

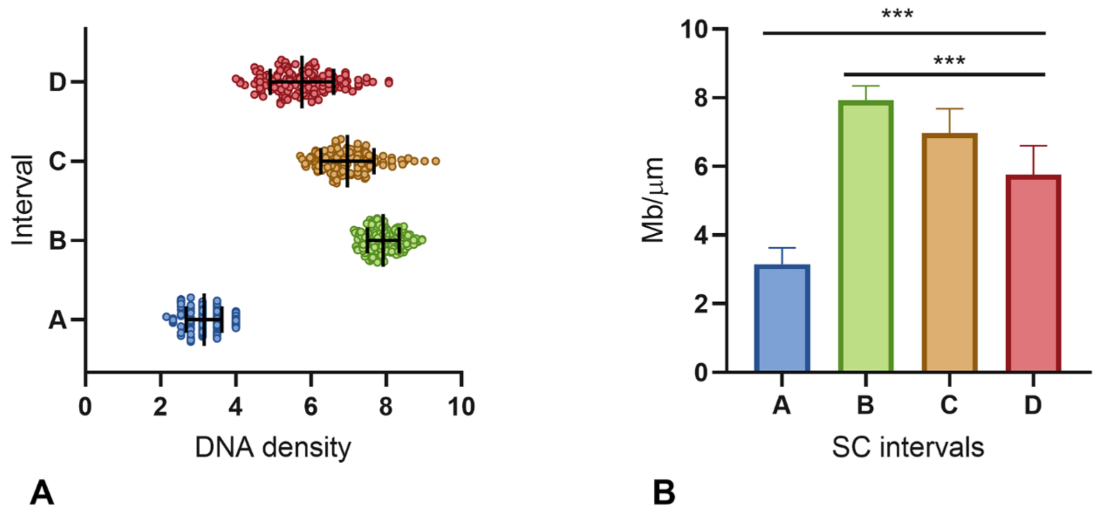

Figure S2. DNA density at different intervals along SC1.

A. DNA density (Mb/µm) for each individual interval generated between the BAC-FISH signals on SC1. The black lines are the mean and the standard deviations.   **B**. Each column represents the average DNA density in the four intervals (A, B, C and D) which are delimited by the five BAC clones used for FISH. There are statistically significant differences between the mean DNA densities, as determined by one-way ANOVA [F (3,

570) = 1339 ; P<0.0001)]. The means are also statistically different if the SC/DNA ratio if data for interval A are excluded from the analysis [(F (2, 473) = 445,8; P<0.0001)].

Data values for Figure 4.

Table S2. DNA density in the intervals limited by the BAC clone positions on the SC.

Intervals between BACs		Interval size			DNA density	Difference [c]
**BACs**	Interval [a]	µm	bp	Mb [b]	Mb/µm	%
**40N14-135N20**	A	0.9	2,795,542	2.8	3.3	-51.4
**135N20-174B20**	B	4.4	35,145,533	35.1	7.9	16.2
**174B20-130B17**	C	3.8	25,769,213	25.8	6.7	-1.4
**130B17-162G5**	D	2.5	14,469,507	14.5	5.8	-14.7

[a] Name of the interval
[b] Amount of linear DNA in Mb between the mid-point of adjacent BACs
[c] Difference = [(DNA density in interval / average DNA density) – 1] x 100.

Measurements of FISH signals on SC1

The distance between the end of the short arm of SC1 and each FISH signal was measured using the program Micromeasure 3.0. For each measured nucleus the program gives an Excel file with the relative and absolute distances. Data for each probe are graphically represented in Figure S2. The individual measurements can be found in the deposited research data (Mendeley Data, V1, doi: 10.17632/w3n9xp5dnp.1).

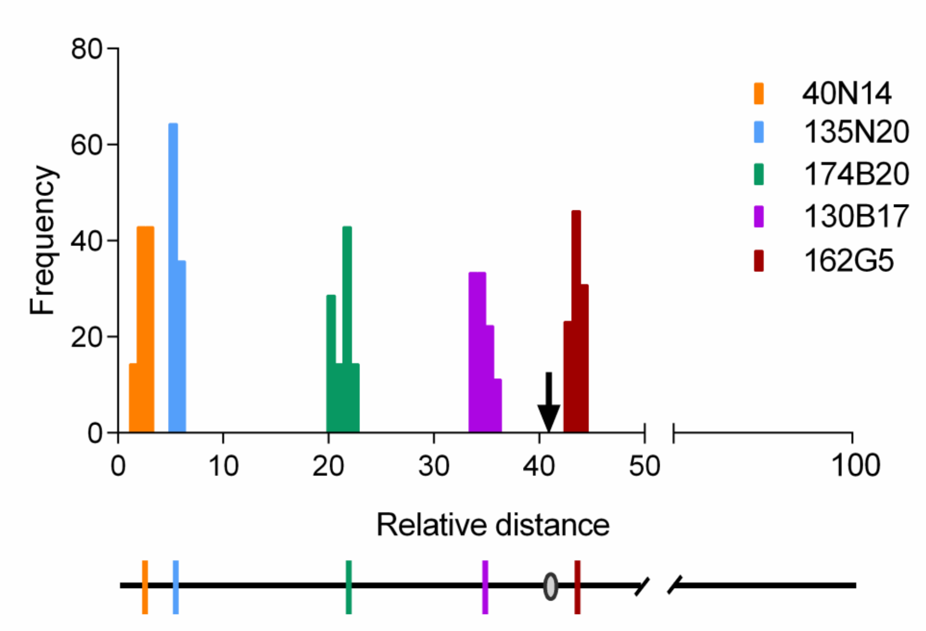

Figure S3. Relative distances of the BAC clones on SC1.

Graphic representation of the distances between the SC term and each FISH signal. The *X* axis is the relative distance as a percentage of the SC length and the Y axis is the percentage of the distances that falls within each interval. The width of each histogram is representative of the dispersion of the FISH signals for individual BACs. The standard deviation of the FISH signals was 0.5 µm or less. The arrow marks the centromere position. Below the graph is a representation of the SC, with the average position of each BAC signal.

Crossover mapping on SC1 and MLH1 counts on macro and microSCs

Crossovers were detected cytologically using an antibody against the protein MLH1 that forms “foci” on the synaptonemal complexes at the sites of crossing over. The distances from the centromere to each MLH1 foci were measured on digitized images of 138 pachytene nucleus to build a histogram of crossover frequencies along SC1 (Figure 5 in the main text).

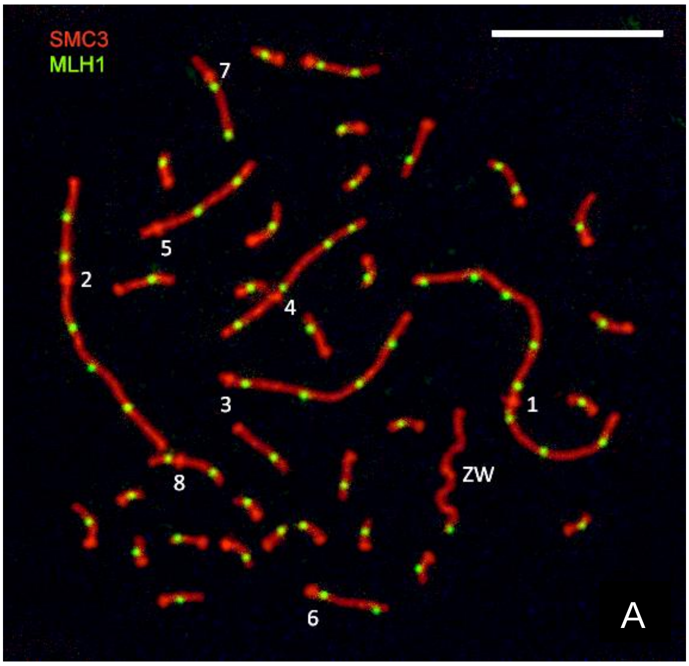

Figure S4. Spread chicken oocytes immunostained for SCs and MLH1. (A). The SCs are labeled with anti-SMC3. The MLH1 foci mark the sites of crossovers. In this nucleus, the SC1 has three foci on the short arm and five of the long arm. The numbers corresponding to the eight largest macro-SCs are next to the centromere signals that are observed as red bulging dots along the SCs. **(B)** Additional images of typical nuclei used for SC measurements and MLH1 focus counts. The bar represents 10 μm.

Crossover mapping on SC1 and MLH1 counts on macro and microSCs *(continued)*

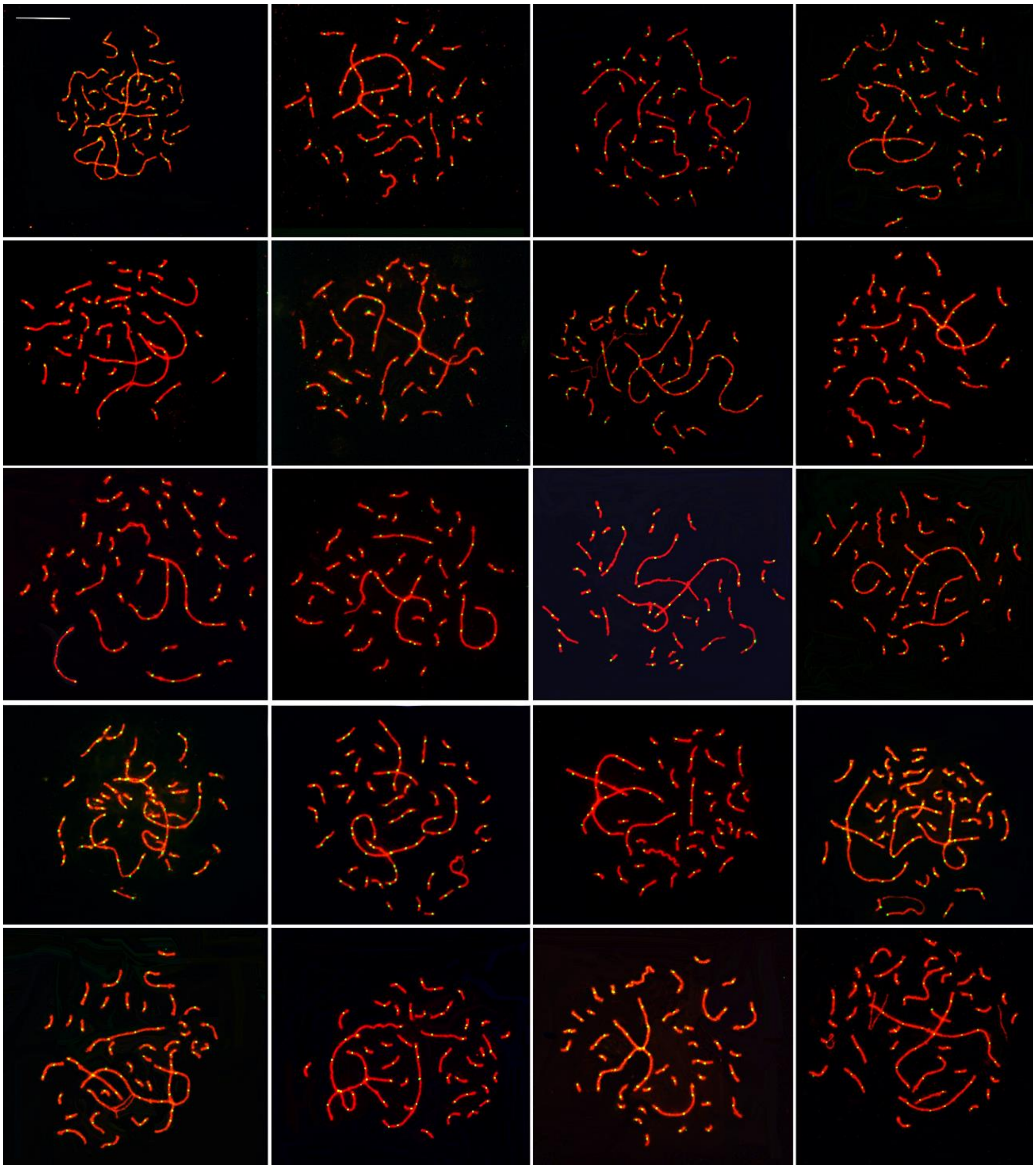

Figure S5. Additional typical pachytene nuclei used for MLH1 focus counts

GC content profile

The GC content profile along GGA1 was obtained using Emboss Isochore (https://www.bioinformatics.nl/cgi-bin/emboss/help/isochore). Windows were set at 1.7 Mb to match the approximate size of the bins in the crossover distribution (Figure 5, main text). The Isochore output file was used to create a G+C plot (Figure S6) comprising the first 80 Mbp of the chromosome sequence.

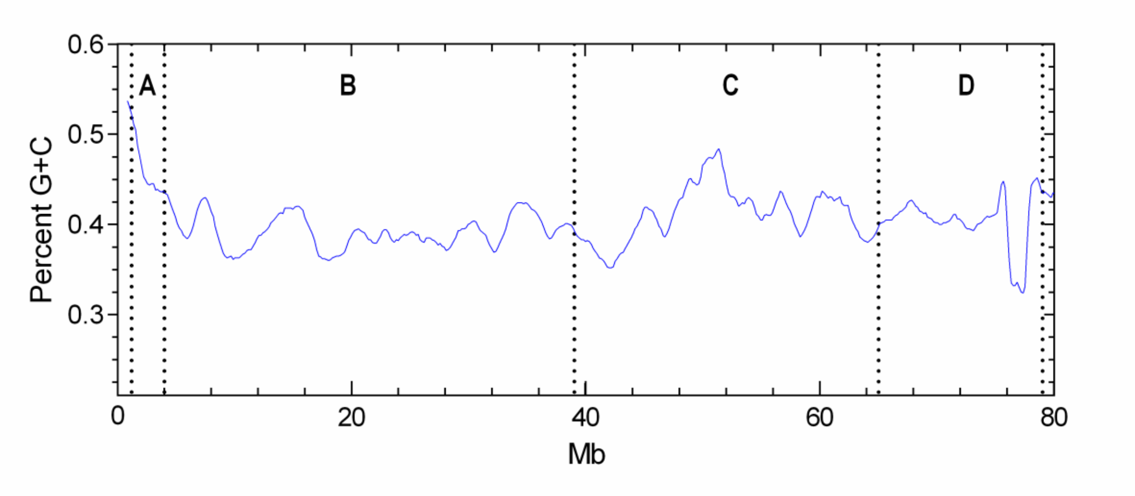

Figure S6. GC content plot of GGA1 1-80.

G + C content of the region 1-80 Mb of GGA1. The segmented lines mark the intervals between the positions of the BAC clones. The average GC content is higher in interval A (46%), compared to the other intervals (39-41%). The drop in interval D correspond to the putative position of the centromere in the GGA1 assembly. Data for this graph are in Mendeley Data, V1, doi: 10.17632/w3n9xp5dnp.1.

Crossover rates in macro and microSCs compared to chromosome size

Table S3. Average number of foci in macro- and microSCs.

Chrom	Foci	cM	Mb	cM/Mb
**1**	7.2	360	197	1.8
**2**	5.9	295	150	2.0
**3**	4.1	205	111	1.8
**4**	3.7	185	91.32	2.0
**5**	2.9	145	59.81	2.4
**6**	1.7	85	36.37	2.3
**7**	1.8	90	36.74	2.4
**8**	1.8	90	30.22	3.0
**microSCs**	1.7[a]	85	14.5	5.8

[a]Foci on microbivalentes 9-38. The number of foci is the average scored in 138 nuclei. Experimental data can be found at Mendeley Data, V1, doi: 10.17632/w3n9xp5dnp.1

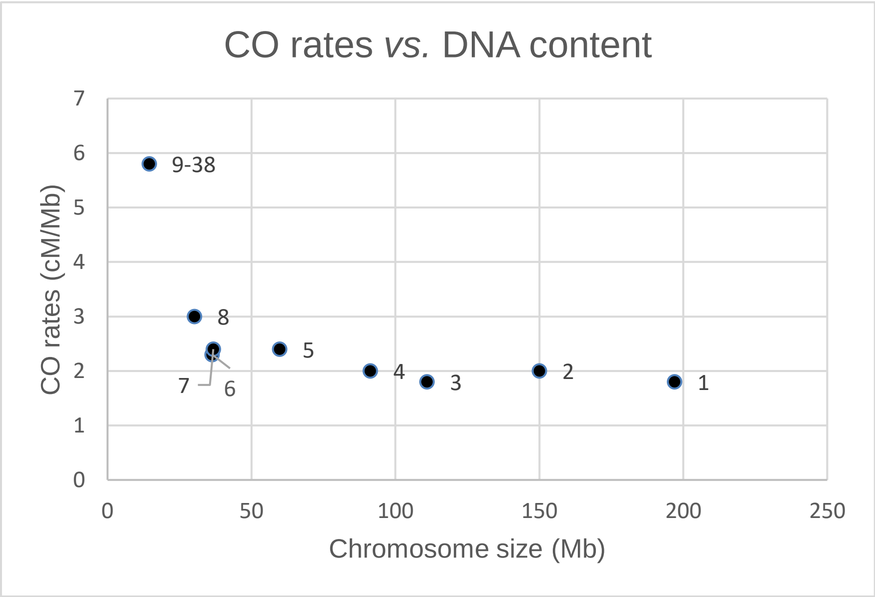

Figure S7. Chromosome size and CO rates are inversely related. Notice the high CO rates of microchromomes (9-38) compared to macrochromosomes (1-8.) The average size of microchromosomes is Total Mb size of microchromosomes (438 Mb) / 30 (microSCs 9-38). Data points are in Table S3. The average size of microchromosomes was used because individual microSCs cannot be assigned with certainty to chromosomes in the assembly. In addition, several microchromosomes are not fully assembled and chromosome 34 to 38 has not been assigned to a linkage group yet Warren et al 2017.
